# Anti-Adenoviral Effect of Human Argonaute 2 Alone and in Combination with Artificial microRNAs

**DOI:** 10.3390/cells13131117

**Published:** 2024-06-28

**Authors:** Philipp Ausserhofer, Izabella Kiss, Angela Witte, Reinhard Klein

**Affiliations:** 1 Institute of Biotechnology, IMC University of Applied Sciences Krems, Piaristengasse 1, 3500 Krems, Austria; philipp.ausserhofer@gmail.com (P.A.);; 2 Center for Pathobiochemistry and Genetics, Medical University of Vienna, Währinger Straße 10, 1090 Vienna, Austria; 3 Department of Microbiology, Immunobiology and Genetics, Max Perutz Labs, University of Vienna, Dr. Bohr-Gasse 9, 1090 Vienna, Austria

**Keywords:** RNA interference, microRNA, AGO2, adenovirus, cellular defense

## Abstract

During infection, adenoviruses inhibit the cellular RNA interference (RNAi) machinery by saturating the RNA-induced silencing complex (RISC) of the host cells with large amounts of virus-derived microRNAs (mivaRNAs) that bind to the key component of the complex, Argonaute 2 (AGO2). In the present study, we investigated AGO2 as a prominent player at the intersection between human adenovirus 5 (HAdV-5) and host cells because of its ability to interfere with the HAdV-5 life cycle. First, the ectopic expression of AGO2 had a detrimental effect on the ability of the virus to replicate. In addition, in silico and in vitro analyses suggested that endogenous microRNAs (miRNAs), particularly hsa-miR-7-5p, have similar effects. This miRNA was found to be able to target the HAdV-5 DNA polymerase mRNA. The inhibitory effect became more pronounced upon overexpression of AGO2, likely due to elevated AGO2 levels, which abolished the competition between cellular miRNAs and mivaRNAs for RISC incorporation. Collectively, our data suggest that endogenous miRNAs would be capable of significantly inhibiting viral replication if adenoviruses had not developed a mechanism to counteract this function. Eventually, AGO2 overexpression-mediated relief of the RISC-saturating action of mivaRNAs strongly enhanced the effectiveness of artificial miRNAs (amiRNAs) directed against the HAdV-5 preterminal protein (pTP) mRNA, suggesting a substantial benefit of co-expressing amiRNAs and AGO2 in RNAi-based strategies for the therapeutic inhibition of adenoviruses.

## 1. Introduction

Human adenoviruses [1,2] are DNA viruses responsible for a wide spectrum of clinical symptoms. Infections primarily manifest in the respiratory and intestinal tracts or eyes and are typically self-limiting. In severe cases, mostly occurring among immunocompromised individuals after organ or stem cell transplantation or as a result of inborn or virus-mediated immune deficiencies, adenovirus infections can become serious and, in some cases, life-threatening [3,4,5,6]. However, therapeutic options are limited, and treatment mostly requires the repurposing of drugs approved for the treatment of other viral infections. This includes primarily nucleoside analogs such as cidofovir (CDV) and its derivatives, all of which are, however, to various extents associated with toxic side effects [7,8,9,10,11].

Human adenoviruses encode one or two virus-associated RNAs (VA RNAs), RNA polymerase III-transcribed, non-coding RNAs approximately 160 nt in length, which are expressed throughout the viral life cycle [12]. While information available on the function of VA RNAII is scarce [13], the function of VA RNAI is better understood. VA RNAI is best known for its ability to bind and inactivate the innate immune response effector protein kinase R (PKR), thereby preventing the otherwise triggered shutdown of protein synthesis upon human adenovirus encounter, which would be detrimental to viral replication (for review, see [12]).

Moreover, Dicer-mediated processing of both VA RNAs I and II gives rise to virus-associated microRNAs (mivaRNAs) that are incorporated into RNA-induced silencing complexes (RISCs), the mediators of microRNA (miRNA)-induced targeting of mRNAs [14,15,16,17,18,19,20,21], and have been shown to target a confined set of cellular transcripts [13,16,22]. Although targeting some of these is conceivably beneficial for the virus, a positive impact on viral replication has only been reported for a single VA RNAII-derived mivaRNA [13]. By contrast, the sequence-specific gene silencing activity of VA RNAI-derived mivaRNAs is dispensable at least during lytic infection under laboratory conditions [23]. However, this does not exclude a possible important function under other conditions or during a non-lytic infection of cells.

Moreover, as mivaRNAs are produced in high quantities, they can partially saturate RISCs [24,25], thereby preventing the efficient incorporation of cellular miRNAs and consequently suppressing the endogenous RNA interference (RNAi) machinery [14,15,16,21], while promoting their own effector functions. In agreement with these findings, a trend toward global derepression of post-transcriptional targeting of cellular mRNAs by endogenous miRNAs appears to occur in adenovirus-infected cells during lytic infection [16]. This effect was found to be most pronounced for RNAs proven to be targeted by cellular miRNAs. The fact that mivaRNAs partially inhibit cellular miRNA function in a sequence-independent manner eventually led to the hypothesis that adenoviruses may benefit from this circumstance. However, this hypothesis has not been tested yet. The deregulation of a large number of cellular regulatory circuits upon mivaRNA-mediated dampening of miRNA-dependent regulation of gene expression and a resulting net benefit for the virus are conceivable. In any case, it will be challenging to prove this net positive effect because of the complex interplay between cellular processes affected by miRNAs and the fact that some miRNAs are antiviral, whereas others are proviral. Previous studies have identified single players in the field, such as hsa-miR-27, which has been described to inhibit viral replication via the suppression of SNAP25 and TXN2 [26], whereas another miRNA, hsa-miR-26b, seems to promote adenoviral replication [27].

However, one of the most likely reasons why adenoviruses have evolved to inhibit cellular RNAi may be to protect their own mRNAs from attack by cellular miRNAs, thereby promoting viral gene expression. Clearly, like their cellular counterparts, viral mRNAs are amenable to targeting by endogenous miRNAs. Hence, in many cases, viruses have developed mechanisms to inhibit the RNAi pathway [28,29]. These include mivaRNA-mediated RNAi impairment by adenoviruses.

In the present study, we aimed to reverse the mivaRNA-induced saturation of RISCs to investigate the role of Argonaute 2 (AGO2) [30], the central and limiting component in RISCs [31], during the human adenovirus 5 (HAdV-5) life cycle in the human cell lines A549 and HeLa and evaluate the impact of endogenous miRNAs on viral replication. This study demonstrates that AGO2 has a detrimental effect on wt HAdV-5 while sparing the VA RNA-deficient HAdV-5 mutant dl-sub720 [32] from a similar effect. Moreover, the anti-adenoviral effect of AGO2 appears to require its slicer function, as respective AGO2 mutants [33] are incapable of inhibiting HAdV-5 replication. Our data suggest at least a dual role for AGO2 besides its involvement in executing mivaRNA sequence-dependent processing of target mRNAs. To highlight the complex interplay between adenoviruses and the human RNAi machinery, we showed that the endogenous miRNA hsa-miR-7-5p is prominently involved in anti-HAdV-5 defense next to other miRNAs, with a less pronounced individual impact. The negative impact of endogenous miRNAs on viral replication becomes especially pronounced upon the reversal of the mivaRNA-induced bottleneck in RISCs by ectopic overexpression of AGO2. This effect occurs not only with cellular viral mRNA-targeting miRNAs such as hsa-miR-7-5p but also with artificial miRNAs (amiRNAs). Thus, we provide a rationale for the additional expression of AGO2 in strategies aimed at the inhibition of adenoviral replication by means of anti-viral amiRNAs, which have been proven to be successful both in vitro and in vivo [34,35,36], and we demonstrate the benefit by using amiRNA/AGO2 co-expression vectors designed to target the adenoviral preterminal protein (pTP) mRNA.

## 2. Materials and Methods

### 2.1. Cell Culture, Virus Amplification, and Titer Determination

HEK-293 (human embryonic kidney; ATCC CRL-1573), A549 (human epithelial lung carcinoma; ATCC CCL-185), HeLa (human epithelial carcinoma; ATCC CCL-2), and HEK-293-TRex cells (Life Technologies Austria, Vienna, Austria) were cultivated in Dulbecco’s Modified Eagles Medium (DMEM) with stabilized glutamine (Gibco, 61965059; ThermoFisher Scientific, Vienna, Austria) and supplemented with 10% fetal bovine serum (FBS; Gibco, 16140071; ThermoFisher Scientific, Vienna, Austria) in a humidified 5% CO_2_ atmosphere at 37 °C. Replication-deficient, E1/E3-deleted, first-generation adenoviral vectors were amplified in HEK-293-TRex cells stably expressing a tetracycline repressor to prevent transgene expression from the TetO2 operator-containing cytomegalovirus (CMV) promoter driving transgene expression. All other adenoviral vectors, wild-type (wt) HAdV-5 (ATCC VR-5) and the adenovirus mutant dl-sub720 [32], were amplified in HEK-293 cells. The mutant virus, dl-sub720, was a gift from Göran Akusjärvi, Department of Medical Biochemistry and Microbiology, Uppsala University, Uppsala, Sweden. The Fast-Trap Virus Purification and Concentration Kit (Merck, FTAV00003, Vienna, Austria) was used for virus purification.

### 2.2. Plasmid and Adenoviral Vector Construction

The constructs employed in the dual-luciferase assays for the evaluation of HAdV-5-targeting amiRNAs and cellular miRNAs have been previously described [34]. Briefly, target sequences of amiRNAs were introduced into the 3′UTR of a Renilla luciferase gene in a psiCHECK-2 dual-luciferase reporter plasmid (Promega, Mannheim, Germany). The firefly luciferase gene present on the same vector remained unchanged and served to normalize the values.

The replication-deficient E1/E3-deleted adenoviral vectors used in this study were based on materials generated in previous work [34,37]. Briefly, historical constructs provided a cassette consisting of a single transcript with a 5′ part coding for EGFP followed by six tandemly arranged amiRNA units targeting HAdV-5 pTP or a respective negative control vector containing six non-targeting control amiRNAs (6xNT). To obtain replication-deficient E1/E3-deleted adenoviral vectors for the co-expression of AGO2/amiRNA, three DNA fragments were combined using Gibson Assembly (NEBuilder HiFi DNA Assembly Master Mix, E2621S; New England Biolabs, Frankfurt am Main, Germany). Fragment 1 (pENTR4 plasmid backbone with regulatory elements: 042_FW 5′- CTAGAGTCCGGAGGCTGGATC -3′, 051_RV 5′- GATCTGGCCGCACTCGAGATTACTGATCAGCCTCGACTGTGCCTTC -3′), fragment 2 (AGO2; template pcDNA4/TO-GFP-hAGO2: 043_FW 5′- GACCGATCCAGCCTCCGGACTCTAGATGTACTCGGGAGCAGGCCCCGCACTTGCACCTCCTGCGCCGCCGCCCCCCATC-3′, 055_RV 5′- TCGACTCACTACCTCCCTTTTTATTAAGCAAAGTACATGGTGCGCAGAGTG-3′), fragment 3 (6xNT or 6xpTPmi5; restriction digest of corresponding, historical plasmid with DraI and EcoRV followed by gel-extraction). Molecular cloning gave rise to the novel plasmids pENTR4[AGO2-6xpTPmi5] and pENTR4[AGO2-6xNT]; negative controls co-expressing EGFP instead of AGO2 were adopted from a previous study without further modifications: pENTR4[EGFP-6xpTPmi5] and pENTR4[EGFP-6xNT] [34].

Constructions of vectors expressing only AGO2 were carried out as follows: The Gibson Assembly was performed with two fragments: fragment 1 (AGO2: 043_FW 5′- GACCGATCCAGCC-TCCGGACTCTAGATGTACTCGGGAGCAGGCCCCGCACTTGCACCTCCTGCGCCGCCGCCCCCCATC-3′, 020_RV 5′-GATCAGTAATCTCGAGTGCGGCCTCGCGAAGCAGCGCAAAACG-3′), fragment 2 (pENTR4 plasmid backbone: 042_FW 5′- CTAGAGTCCGGAGGCTGGATC-3′, 015_RV 5′- GGCCGCACTCGAGATTACTGATCAGCCTCGACTGTGCCTTC-3′). A corresponding negative control vector containing pCMV-TetO2-EGFP was previously established [34].

**Figure 1:**
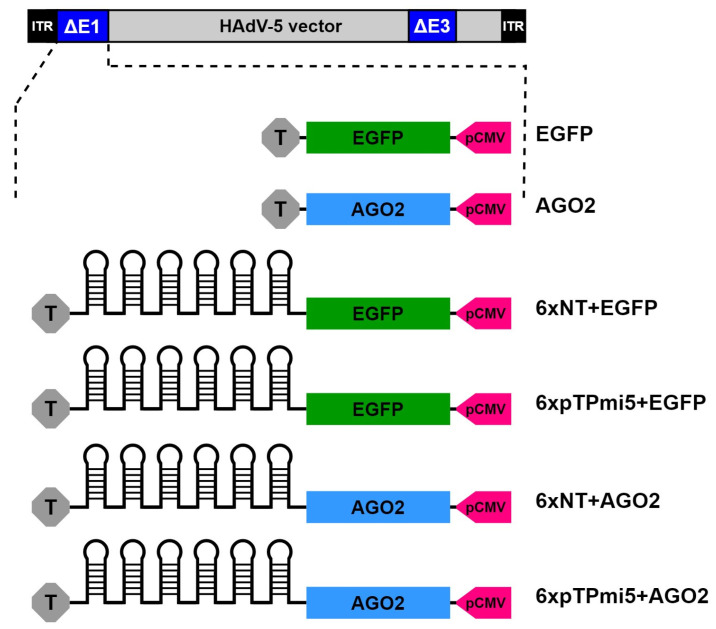
Schematic representation of the adenoviral vectors used in this study. All vectors were based on the HAd5V-derived vectors pAd/PL-DEST ™ (ThermoFisher Scientific, Vienna, Austria) lacking the E1 and E3 genes. Expression cassettes were inserted into the deleted E1 region in antisense orientation with respect to the left inverted terminal repeat (ITR). The expression cassettes contain EGFP [34] or AGO2 (this study) open reading frames, either alone or in conjunction with six tandemly repeated, either targeting (pTP-mi5) or non-targeting (NT), amiRNA hairpins incorporated into the 3′ UTR of the EGFP and AGO2 transcripts, respectively. Expression is driven by a CMV promoter (pCMV).

Generation of adenoviral pAd/PL-DEST vectors: The intermediate pENTR4-based expression cassettes were transferred into pAd/PL-DEST plasmids (Life Technologies Austria, Vienna, Austria) by site-specific recombination using Life Technologies’ Gateway technology, as described by the manufacturer. Eventually, the PacI-digested linear pAd/PL-DEST destination vectors were transfected into HEK-293-TRex cells to enable the formation of infectious recombinant adenovirus vectors for all further experiments. All adenoviral vectors used in this study are depicted in Figure 1. Plasmids containing the AGO2 mutants, D597A or PAZ10, were obtained from Greg Hannon (University of Cambridge, Cambridge, UK).

Restriction enzymes and DNA-modifying enzymes were purchased from New England Biolabs (Frankfurt am Main, Germany). PCR reactions were performed with Q5^®^ Hot Start High-Fidelity Polymerase (NEB M0494S, New England Biolabs, Frankfurt am Main, Germany). Gel extraction was performed using a QIAquick Gel Extraction Kit (QIAGEN 28704; QIAGEN, Hilden, Germany). Transfection was performed using Lipofectamine 2000 (Life Technologies Austria, Vienna, Austria).

### 2.3. Nucleic Acid Extraction

Circular plasmid DNA was extracted from bacteria using a QIAGEN Plasmid Midi Kit (QIAGEN, Hilden, Germany). PCR products were purified using a QIAquick PCR Purification Kit (QIAGEN, Hilden, Germany) or, when required, gel-extracted (QIAGEN, Hilden, Germany). Adenoviral DNA was isolated from eukaryotic cells using a QIAamp DNA Blood Mini Kit (QIAGEN, Hilden, Germany).

### 2.4. Dual-Luciferase Assays

To test the capacity of individual miRNA mimics to silence the respective psiCHECK-2-derived mRNA targets (Pol, pTP), 1.5 × 10^4^ HeLa cells were seeded into 96-well plates and transfected with 100 ng of individual dual-luciferase reporter vectors and 10 nM miRNA mimics using Lipofectamine 2000 (Life Technologies Austria, Vienna, Austria). Firefly and Renilla luciferase activities were measured at 48 h post-transfection using the Dual-Glo Luciferase Assay Kit (Promega, Mannheim, Germany; E1910) according to the manufacturer’s instructions. Briefly, knockdown rates were calculated by normalizing Renilla luciferase to firefly luciferase values, followed by a comparison of dual-luciferase ratios between the individual miRNA mimics and the non-targeting negative control (NT).

Likewise, the individual and combined action of miRNA mimics and Power Inhibitors (PI) was investigated by co-transfecting 1.5 × 10^4^ HeLa cells with 100 ng dual-luciferase reporter vectors (Pol) and miRNA mimics and/or PI at 5 nM, respectively. A single non-targeting control (NT) species was used for either nucleic acid class as per the instructions of the manufacturer. Owing to the experimental design, the total concentration of transfected RNA reached 10 nM in each condition. The luciferase readout, as per the instructions of the manufacturer, was followed at 48 h post-transfection.

### 2.5. Virus Inhibition Experiments

Inhibition experiments based on plasmid-encoded anti-adenoviral elements were conducted as follows: 1.5 × 10^4^ HeLa or A549 cells were seeded into 96-well plates 24 h prior to simultaneous transfection (Lipofectamine 2000; Life Technologies Austria, Vienna, Austria) with 250 ng plasmid DNA and infection with wt HAdV-5 or mutant virus dl-sub720 at an MOI of 0.1 TCID50/mL. Viral genome copy numbers were determined at 96 h post-infection.

Inhibition experiments based on replication-deficient adenoviral vector—located elements: 1.5 × 10^4^ HeLa or A549 cells were seeded into 96-well plates for 24 h before being transduced with vectors at an MOI of 100/cell. During prophylactic scenarios, wt HAdV-5 was added 24 h later at an MOI of 0.1 TCID50/cell while cells were simultaneously transduced and infected in the therapeutic approach.

For experiments involving miRNA mimics, (i) 1.5 × 10^4^ HeLa cells were simultaneously transfected (5 nM) and transduced with adenoviral vectors at an MOI of 100 TCID50/mL. HAdV-5 was added 24 h later at an MOI of 0.1 TCID50/mL. Additional formats included (ii) simultaneous transfection with miRNA mimics at 10 nM and infection with HAdV-5 at an MOI of 0.1 TCID50/mL; (iii) co-transfecting miRNA mimics at 10 nM and dual-luciferase reporter vectors at 100 ng; and (iv) co-transfecting combinations of miRNA mimics and PIs at 2 × 5 nM, each, in addition to the 100 ng dual-luciferase reporter vector.

Two-dimensional vector/wt HAdV-5 matrices were prepared following the abovementioned therapeutic procedures involving simultaneous transduction and infection. Briefly, the adenoviral vector was added according to the indicated MOI gradient (50, 100, 250, 500, and 1000 TCID50/cell), and each condition was separately combined with HAdV-5 at MOIs of 0.01, 0.1, 1, 10, and 100 TCID50/cell.

For co-transduction of replication-deficient adenoviral vectors, 1.5 × 10^4^ A549 cells were cultivated for 24 h before the vectors were added at 250 TCID50/mL. Wt HAdV-5 was added 24 h later at an MOI of 1 TCID50/cell, and the samples were harvested 72 h later.

For downstream analytical methods such as quantification of genome copy numbers/mL and/or TCID50/mL, plates were harvested at the indicated timepoints and stored at −80 °C. Crude viral suspensions were obtained by freezing/thawing the plates thrice.

### 2.6. Determination of Adenovirus Genome Copy Numbers

Wt HAdV-5 DNA genome concentration (genome copy numbers/mL) was determined by qPCR using the following TaqMan FW-Primer/RV-Primer/Probe set directed against the viral E3 region: E3_FW 5′-TGCTGCACTGCTATGCTAAT-3′, E3_RV 5′TCCTCAATAAAGCTGCGTCTG-3′, and E3_P 5′-TGCTCGCTTTGGTCTGTACCCTAC-3′. Genome copy number concentrations were calculated using serial dilutions of an adenoviral reference DNA as a standard. Genome abundance of amiRNA-expressing recombinant viruses was determined using a TaqMan primer/probe set specific for either the adenoviral hexon gene (Hexon_FW 5′-CACTCATATTTCTTACATGCCCACTATT-3′, Hexon_RV 5′-GGCCTGTTGGGCATAGATTG-3′, Hexon_P 5′-AGGAAGGTA ACTCACGAGAACTAATGGGCCA-3′) when cells had not concomitantly been infected with wt HAdV-5 or the vectors’ immediate early CMV promoter region (CMV_FW 5′- GGTGGAGACTTGGAAATC-3′, CMV_RV 5′- GTCAATGACGGTAAATGG-3′, CMV_P 5′- CAAGTAGGAAAGTCCCATAAGGTCA-3′) when cells had concomitantly been infected with wt HAdV-5. For readout, a Biozym Blue Probe qPCR Separate ROX kit (Biozym 331456XL; Biozym, Vienna, Austria) was used. For data acquisition and analysis, the QuantStudioTM 7 Flex System (1 × 2′-50 °C, 1 × 5′-95 °C, 40 × 15″-95 °C/1′-60 °C) was used.

### 2.7. Determination of Adenovirus Infectious Particle Numbers

Samples (mammalian cell culture with either wt HAdV-5 alone or in combination with recombinant HAdV-5) underwent three frost/thaw cycles before being analyzed with an Adeno-X™ Rapid Titer Kit (632250, Takara, Göteborg, Sweden). Briefly, 1.25 × 10^5^ HeLa cells were simultaneously seeded into 24-well plates and infected with 50 μL of virus suspension of interest. Readout occurred at 48 h post-exposure by immunocytochemical detection of the late adenoviral hexon protein. HAdV5-positive cells were counted, and the counts were converted into infectious titers (TCID50/mL).

### 2.8. Bioinformatic ANALYSIS

Putative miRNA-binding sites were predicted using TargetScan 7.0 [38]. The Perl scripts for custom datasets were downloaded from http://www.targetscan.org (accessed on 5 February 2020). A list of high-abundance miRNAs in HAdV-5-infected human A549 cells derived from previous NGS data [16] was used as a query for the genome of human adenovirus C serotype 5 (GenBank: AY339865.1). miRNA sequences were extracted from miRBase (http://www.mirbase.org (accessed on 5 February 2020) [39]. Adaptions to account for the custom input data set as per correspondence with TargetScan creators were as follows: The intermediate step of calculating the “probability of conserved targeting” (PCT) values using the script package targetscan_70_BL_PCT.zip was entirely omitted. Instead, the output table of the first script package targetscan_70.zip was modified by keeping columns 1–11 and introducing two additional columns with constant values (branch length score = 0; PCT = NA). This file was then fed into the third script package, TargetScan7_context_scores.zip. Two of the five input files remained empty (ORF_Sequences_sample.lengths.txt and ORF_8mer_counts_sample.txt). Part of the custom input files’ nomenclature had to be adjusted to obtain a productive outcome: “has-miR” had to be changed to “miR”. The output file was modified as follows: the column ORF length contribution was set to zero, and a custom Context Score was calculated by adding up values from columns 7–27. Only matches with perfect seed sequence complementarity were considered (8mer-1A and 7mer-m8). Hits were assigned to HAdV-5 mRNAs E1A-27 kDa, E1A-32 kDa, E1B, E2B-Pol, E2B-pTP, IX, E3, and E4 and sorted by context score, where low scores were considered favorable.

### 2.9. miRNA Mimics

To proceed with a potentially promising subset of putative anti-adenoviral miRNAs, we assessed the TargetScan 7.0 output according to the following criteria: read count, context score, binding site frequency per transcript, shared binding sites, binding site clusters, and targeting by 5p and 3p of the same mRNA species. As a result, we focused on 11 miRNA mimics to proceed with (Table 1). The respective duplexes were generated and provided by Biomers (Ulm, Germany). The integrity of the mimic duplexes was verified by loading samples onto a 5% agarose gel. Lyophilized, 5 nmol of mimics were resuspended in 500 μL nuclease-free water to yield a 10 μM stock from which 800 nM stock solutions were prepared for experiments. Solutions were stored at −80 °C.

### 2.10. Power Inhibitors

A representative subset of promising miRNAs was selected for further analyses. Bespoke Power Inhibitors (PIs; miRCURY LNA miRNA Power Inhibitors; QIAGEN, Hilden, Germany), functioning as antagomirs with full complementarity to their miRNA targets) were designed by QIAGEN based on a provided list of miRNAs of interest. Lyophilized, 1 nmol PIs were resuspended in 100 μL nuclease-free water to obtain a 10 μM stock from which 800 nM stock solutions were prepared for experiments. Solutions were stored at −80 °C.

### 2.11. Western Blotting

Proteins were separated by sodium dodecyl sulfate-polyacrylamide gel electrophoresis on Mini-Protean TGX Precast protein gels (Bio-Rad, Hercules, CA, USA) and transferred onto nitrocellulose membranes (Bio-Rad) using a Turbo transfer system (Bio-Rad). Membranes were blocked with 5% BSA T-BST (500 mM Tris HCl [pH 7.5], 1.5 M NaCl, 0.05% Tween 20). Human AGO2 and β-actin were detected with the antibodies mouse anti-panAGO (Clone 2A8, Lot# 2146028, CAT# MABE56, Merck/Millipore, Vienna, Austria) and rabbit #4970 (Cell Signaling Technology, Danvers, MA, USA), respectively. Membranes were probed with the fluorescent secondary antibodies IRDye 800CW goat anti-mouse (925-32210, LI-COR Biosciences, Lincoln, NE, USA) and IRDye 680RD goat anti-rabbit (925-68071, LI-COR Biosciences), respectively, and bands were visualized with a ChemiDoc MP Imaging System (Bio-Rad, Hercules, CA, USA).

### 2.12. Statistical Analysis

Data are presented as mean ± standard deviation (SD). Student’s *t*-test, one-way analysis of variance (ANOVA), or two-way ANOVA (General Linear Model) were applied to test for statistical significance. When corrections for multiple testing were performed to compare multiple samples with each other or with a single control, Tukey´s and Dunnett’s tests were employed, respectively. P-values were visualized as follows: *p* < 0.05 (*), *p* < 0.01 (**), *p* < 0.001 (***), and *p* < 0.0001 (****), not significant (ns).

## 3. Results

### 3.1. Ectopic Expression of Human AGO2 Impairs the HAdV-5 Life Cycle

We hypothesized that resolving the bottleneck in functional RISC availability during HAdV-5 infection would be detrimental to the virus because of the well-documented saturation of this system with mivaRNAs. Consequently, we ectopically overexpressed AGO2, the limiting factor in RISCs, to allow cellular miRNAs to regain full access to the RNAi machinery.

**Figure 2:**
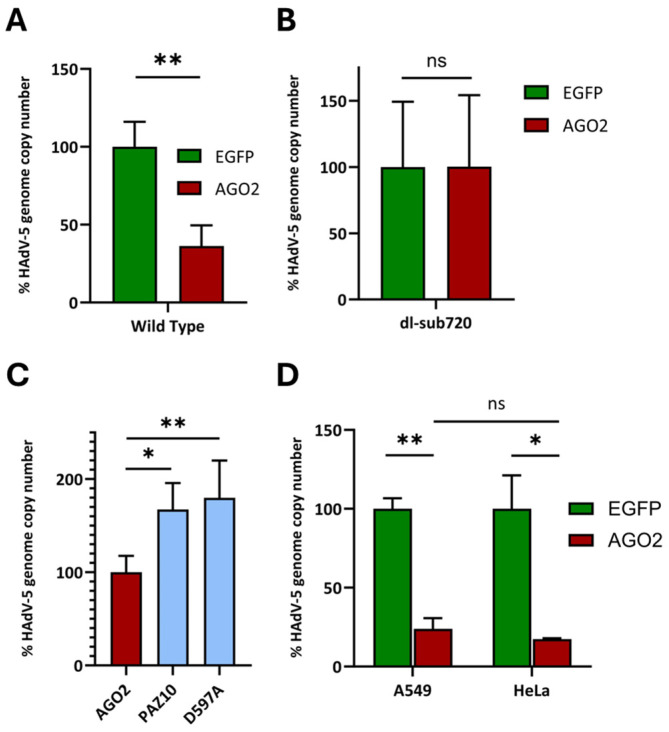
AGO2 negatively affects the HAdV-5 life cycle. (**A**) Overexpression of AGO2 decreases the wt HAdV-5 genome copy numbers. 1.5 × 10^4^ HeLa cells were simultaneously transfected with 250 ng plasmid and infected with wt HAdV-5 at an MOI of 0.1, respectively. Concentrations of HAdV-5 genome copy numbers were measured at 96 h post-transfection/infection using an E3-probe qPCR. The data display means and standard deviations from a total of 3 independent experiments. Each independent experiment’s EGFP control preparation measurement was set as a reference at 100%. ** (*p* < 0.01). (**B**) Overexpression of AGO2 has no effect on the adenovirus mutant dl-sub720. Same as in (**A**), except that cells were infected with the HAdV-5 mutant dl-sub720 instead of wt HAdV-5. ns (not significant). (**C**) Compared to wt AGO2 mutants defective for miRNA binding, they differ in their ability to decrease wt HAdV-5 genome copy numbers. 1.5 × 10^4^ HeLa cells were simultaneously transfected with 250 ng plasmid expressing either AGO2 or mutants thereof and were infected with wt HAdV-5 at an MOI of 0.1. Concentrations of HAdV-5 genome copy numbers were measured at 96 h post-transfection/infection using an E3-probe qPCR. The data represent the means of 3 representative experiments, including standard deviations. The mean value for the AGO2 measurements was set as a reference at 100%. * (*p* < 0.05); ** (*p* < 0.01). (**D**) The inhibition of HAdV-5 replication by AGO2 is comparable in A549 and HeLa cells. 1.5 × 10^4^ A549 or HeLa cells were transduced with AGO2-expressing rAdV vectors at an MOI of 100. 12 h after transduction wt HAdV-5 was added at an MOI of 0.1. Concentrations of HAdV-5 genome copy numbers were measured at 48 h post-infection using an E3-probe qPCR. The data were derived from a total of 3 representative experiments and display mean ± standard deviations. Each experiment’s EGFP control preparation measurement was set as a reference at 100%. * (*p* < 0.05); ** (*p* < 0.01); ns (not significant).

We observed that plasmid-based overexpression of AGO2 had a significant detrimental effect on wt HAdV-5 propagation, based on genome copy numbers (Figure 2A). In contrast, the dl-sub720 viral mutant lacking the VA-RNA I + II locus was unaffected by excess AGO2 (Figure 2B). Concerning wt HAdV-5, the observable effect was reversible when wt AGO2 was replaced by the slicing-deficient AGO2 mutants D597A or PAZ10 (carrying the point mutations R277A, K278A, Y279A, F294A, Y311A, F312G, T337A, Y338A, L339A, and H271A) [33] (Figure 2C). The decrease in viral genome copy number upon AGO2 overexpression was also reflected in a decreased number of infectious virus particles (Figure S1). Eventually, replication-deficient, E1/E3-deleted recombinant adenoviral (rAdV) vector-delivered AGO2 (Figure 1) likewise mediated a robust downregulation of wt HAdV-5 genome abundance, and the effect occurred in both HeLa and A549 cells to a comparable extent (Figure 2D). Downregulation of viral genome copy numbers was observed across a multitude of vector MOIs (Figure S2A), independent vector preparations (Figure S2A), and different transduction/infection scenarios (Figure S2B).

### 3.2. In Silico Analysis Identifies Putative, Cellular, and Anti-Adenoviral miRNAs

Having demonstrated that ectopic expression of AGO2 impairs wt HAdV-5 replication, we hypothesized that de-repression of mivaRNA-saturated RISCs by decoy AGO2 may reinstate antiviral, host-cell miRNA-mediated responses. Using TargetScan 7.0 [38], we searched for conserved 8mer, 7mer, and 6mer sites in HAdV-5 mRNAs that matched the seed sequences of cellular miRNA queries. Specifically, we searched for putative miRNA-binding sites in adenoviral early mRNA sequences covering the E1, E2, E3, E4, and IX transcript families [40]. Late transcripts were not considered because of their high abundance, which was expected to overwhelm the endogenous RNAi capacity. Conversely, the RNAi-mediated elimination of target RNAs should be more pronounced for relatively low-abundance early transcripts. Eventually, a decision was made to only pursue hits associated with E2B transcripts coding for the preterminal protein (pTP) and the DNA polymerase (Pol), which are among the lowest by means of abundance [40,41] and whose targeting by artificial siRNAs had earlier been found by us to result in the most significant negative impact on virus replication [42].

**Figure 3:**
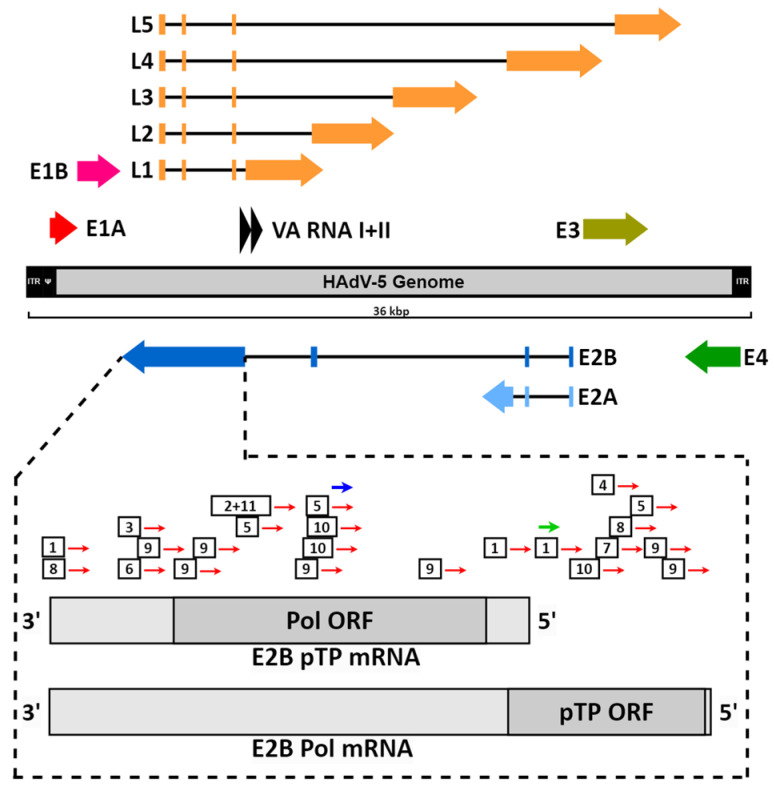
Putative target sites of cellular miRNAs within HAdV-5 early mRNA transcripts Pol and pTP as per in silico TargetScan analysis. In addition to putative target sites of cellular miRNAs (red arrows), the target sites of the previously described siRNAs Pol-si2 (blue arrow) and pTP-si8 [42] with their corresponding amiRNA pTPmi5 (green arrow) [34] are indicated.

Eleven candidate miRNAs (Figure 3) were selected for further investigation based on parameters such as TargetScan score (Table 1), prevalence in the cell lines used in this study, frequency of predicted target sites, miRNA read count per AGO2 IP-Seq (RISC incorporation) [16], association with predicted miRNA clusters, and coverage of both E2B transcripts. Hits were limited to candidates that displayed perfect seed/mRNA target homology. Hsa-miR-7-5p, -423-5/3p, -151a-5/3p, -29-3p, and -let-7a-5p were predicted to bind to multiple sites across E2B mRNAs, sometimes involving both the 5p and 3p single strands of a miRNA duplex. Hsa-miR-let-7a-5p served as a representative of the much larger hsa-let-7 family of highly abundant miRNAs, whose members are characterized by an identical seed sequence and a variation of only one to three nucleotides across the rest of the molecule. Hsa-miR-100-5p and -99b-5p presumably bind to the same spot and display very high read counts. The latter was also partially applied to hsa-miR-22-3p and 27b-3p. Hsa-miR-125b-5p, -29a-3p, -1307-5p, and -7-5p were all characterized by favorable TargetScan scores (Table 1).

### 3.3. Human miRNA-7 Impairs the wt HAdV-5 Life Cycle

**Figure 4:**
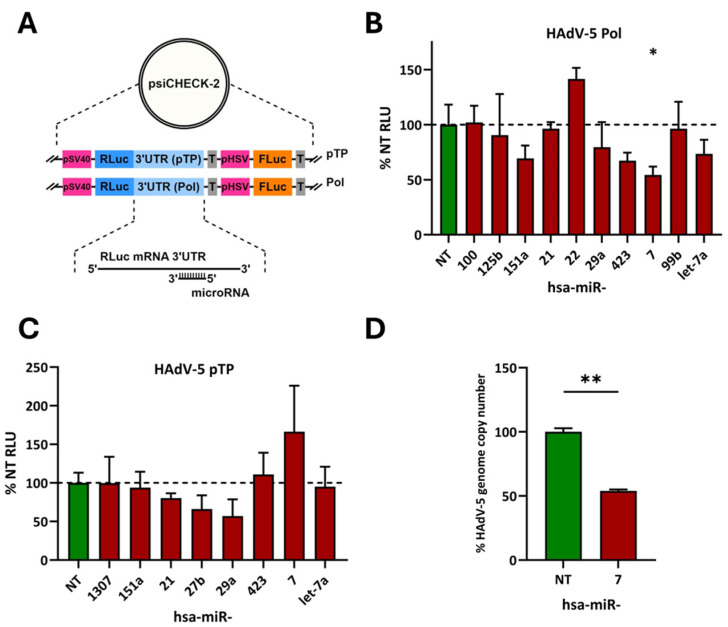
Targeting of HAdV-5 DNA polymerase and pTP sequences by cellular miRNAs and their impact on viral replication. (**A**) Schematic representation of the dual-luciferase reporter vector system employed in this study. Reporter vectors contain sequences of the HAdV-5 DNA polymerase and pTP genes (Pol; pTP) inserted into the 3′UTR of a Renilla luciferase reporter gene (RLuc). MiRNAs capable of recognizing the respective target mRNAs are expected to knock down Renilla luciferase expression relative to the expression of a non-targeted firefly luciferase gene (FLuc) present on the same vector. (**B**) Targeting of the HAdV-5 DNA polymerase mRNA by miRNA mimics in reporter assays. 1.5 × 10^4^ HeLa cells were simultaneously transfected with a HAdV-5 DNA polymerase reporter vector carrying the DNA polymerase sequence inserted into the 3′UTR of a Renilla luciferase reporter gene and endogenous miRNA mimics, respectively. A non-targeting (NT) miRNA mimic was used as a control. Readout as per manufacturers’ instructions was conducted at 48 h post-transfection and relative light units (RLUs) for the Renilla luciferase reporter gene were normalized to those of the firefly luciferase reporter gene. Each experiment’s NT control preparation measurement was set as a reference at 100%. The data represent means ± standard deviation of 3 experiments. * (*p* < 0.05). (**C**) Targeting of the HAdV-5 pTP mRNA by miRNA mimics in reporter assays. Same as in (**B**) except that a reporter vector carrying the HAdV-5 pTP sequence was used. (**D**) Effect of the hsa-miR-7 mimic on HAdV-5 genome copy numbers. 1.5 × 10^4^ HeLa cells were simultaneously transfected with an hsa-miR-7 mimic at a concentration of 10 nM and infected with HAdV-5 at an MOI of 0.1. Concentrations of HAdV-5 genome copy numbers were measured at 48 h post-transfection/infection using an E3-probe qPCR. Data represent mean ± standard deviations of a representative experiment carried out in triplicate. The non-targeting (NT) control preparation measurement was set as a reference at 100%. ** (*p* < 0.01).

Candidate miRNAs, as per TargetScan analysis (Table 1), were further investigated using the miRNA mimic format. To prove that the viral DNA polymerase and pTP sequences are indeed targets of the suspected miRNAs and to avoid possible indirect effects that may be related to altered adenoviral promoter activities occurring as a consequence of miRNA-mediated targeting of other cellular or viral transcripts, we removed the DNA polymerase and pTP sequences from the context of the viral genome and analyzed them separately in reporter assays. To this end, we employed dual-luciferase reporter vectors carrying the viral DNA polymerase or pTP target sequences inserted into the 3′UTR of a Renilla luciferase reporter gene (Figure 4A) [42]. Targeting these transcripts by miRNA mimics is expected to decrease the Renilla luciferase signal relative to the firefly luciferase signal originating from the expression of the respective gene present on the same vectors for normalization purposes.

Co-transfection of cells with the vectors and individual miRNA mimics revealed that the Renilla luciferase signal of the DNA polymerase reporter was significantly reduced upon transfection with hsa-miR-7-5p (Figure 4B), compared to a non-targeting control miRNA. In contrast, the pTP reporter did not react to hsa-miR-7-5p (Figure 4C). Hsa-miR-423-5/3p, -151a-5/3p, -29a-3p, and -let-7a-5p tended to reduce the Renilla luciferase signal level of the DNA polymerase reporter to a well-detectable degree, while the factual readout remained non-significant. This overlapped with the observation that hsa-miR-29a-3p performed best in downregulating the Renilla luciferase signal in the pTP reporter, yet statistically not significantly.

As the most pronounced effect was induced by hsa-miR-7, we consequently tested this miRNA for its ability to impair the life cycle of infectious wt HAdV-5. Upon transfection with the specific hsa-miR-7 mimic, the wt HAdV-5 genome copy number was in fact reduced by approximately two-fold compared to that of the control, thereby confirming the dual-luciferase assay data (Figure 4D).

To further confirm the dual-luciferase and viral inhibition assay data, we introduced Power Inhibitors (PIs). These are oligonucleotides that selectively bind and inhibit either the one (5p) or the other (3p) single strand of a double-stranded miRNA. We used PIs binding to those miRNA single strands that were predicted to target the respective sites in the HAdV-5 transcript. For hsa-miR-423, we used two different inhibitors specific for either the 5p or 3p strands of the miRNA, as both strands have predicted targeting capability.

**Figure 5:**
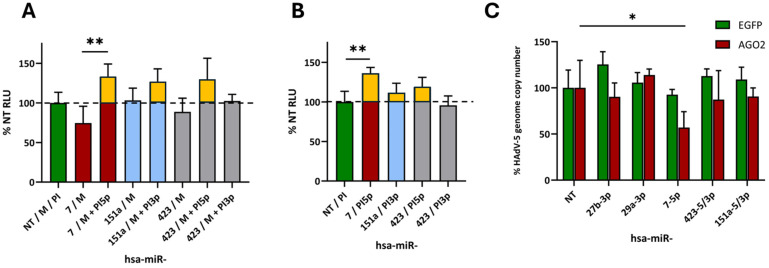
Inhibition of miRNA function with miRNA inhibitors and enhancement of miRNA-mediated effects on HAdV-5 by overexpression of AGO2. (**A**) Inhibition of the action of miRNA mimics with miRNA inhibitors in reporter assays. 1.5 × 10^4^ HeLa cells were simultaneously transfected with (i) a HAdV-5 DNA polymerase reporter vector carrying the DNA polymerase sequence inserted into the 3′UTR of a Renilla luciferase reporter gene, (ii) miRNA mimics, and (iii) corresponding miRNA Power Inhibitors (PI). Readout as per manufacturers’ instructions was conducted at 48 h post-transfection. Data (relative light units; RLU) derives from a total of 3 experiments and displays means ± standard deviations. Each experiment’s non-targeting (NT) control preparation measurement was set as a reference at 100%. ** (*p* < 0.01). (**B**) Effect of miRNA inhibitors in the absence of miRNA mimics in reporter assays. Same as in (**A**) except that no miRNA mimics directed against the reporter transcript were employed. ** (*p* < 0.01). (**C**) Effect of miRNA mimics on HAdV-5 replication. 1.5 × 10^4^ HeLa cells were simultaneously transfected with 5 nM miRNA mimics and transduced with an AGO2- or EGFP-expressing adenoviral vector at an MOI of 100. 24 h later, wt HAdV-5 was added at an MOI of 0.1. Concentrations of wt HAdV-5 genome copy numbers were measured at 48 h post-infection using an E3-probe qPCR. Data represent means ± standard deviations of a representative experiment carried out in triplicate. * (*p* < 0.05).

As expected, the hsa-miR-7-5p inhibitor reversed the miRNA mimic-mediated effect on the DNA polymerase transcript in the dual-luciferase reporter assay (Figure 5A). Since the other miRNA mimics were generally less potent than the hsa-miR-7 mimic, the reversal effects of the respective inhibitors were accordingly less pronounced. When applied in the absence of the respective miRNA mimics, the PIs also showed a tendency to increase the DNA polymerase reporter signal in the case of the hsa-miR-7-5p inhibitor, reaching statistical significance (Figure 5B). This can be interpreted as the abolishment of targeting of the DNA polymerase reporter transcript by endogenous hsa-miR-7-5p. This PI-mediated effect could also be seen in the presence of targeting miRNA mimics (Figure 5A), in which the miRNA-targeting PIs actually raised the reporter signals above those seen for cells that had not been treated with an mRNA-targeting miRNA mimic or with an miRNA-targeting PI (yellow parts of the columns in Figure 5A), i.e., cells that still contained endogenous miRNAs targeting the reporter transcript, suggesting that the PIs had abolished the function of both the miRNA mimics and their corresponding endogenous miRNAs. The PI targeting hsa-miR-423-5p showed a trend similar to that of hsa-miR-7-5p, although it was less pronounced. Conversely, PI hsa-miR-423-3p did not have any effect in the presence (Figure 5A) or absence of the respective miRNA mimic hsa-miR-423 (Figure 5B), suggesting the 5p strand, but not the 3p strand of has-miR-423 to cause the observed downregulation.

### 3.4. AGO2 Co-Expression Improves the Performance of Anti-Adenoviral miRNAs

We hypothesized that the AGO2 overexpression-mediated abolishment of functional RISC shortages caused by mivaRNAs should improve endogenous miRNA effector functions. Indeed, our data indicated that miRNA mimic activity was boosted by the co-expression of AGO2, resulting in a significant impairment of viral replication compared to the EGFP control (Figure 5C).

**Figure 6:**
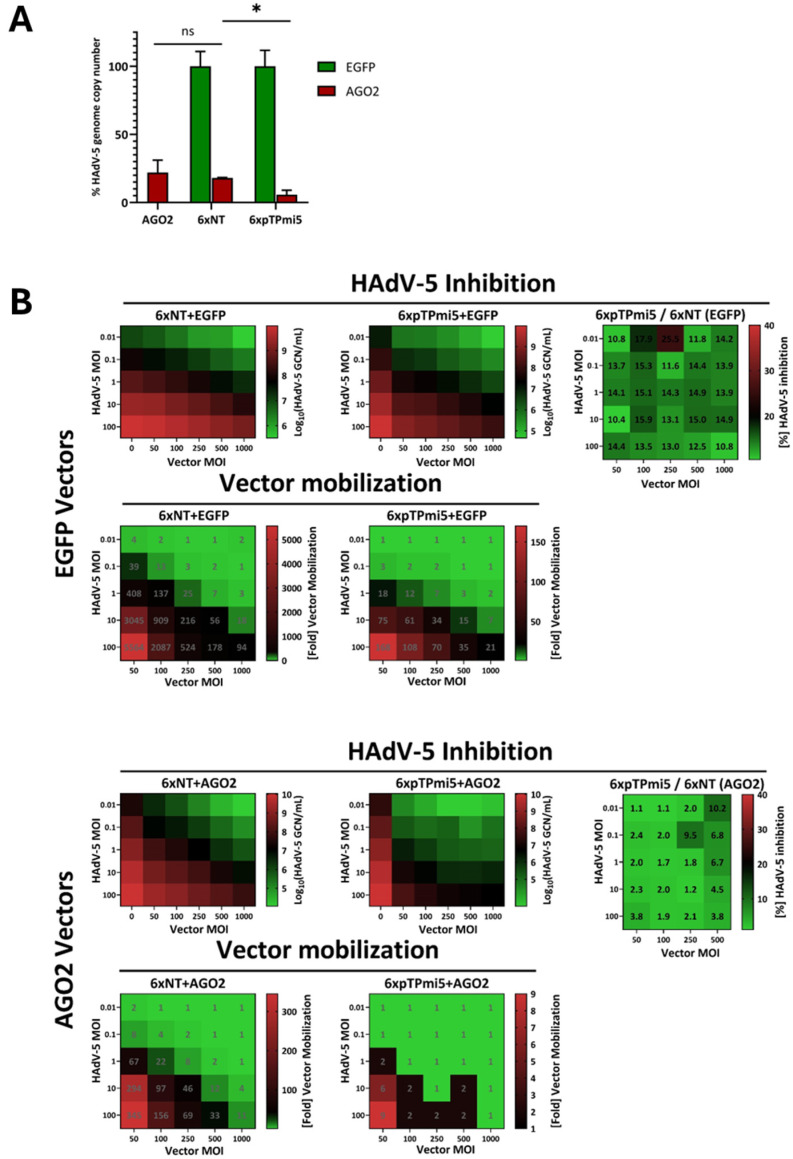
Improved amiRNA-mediated inhibition of wt HAdV-5 replication upon co-transduction with an AGO2-expressing rAdV vector. (**A**) The potency of an amiRNA targeting the adenoviral pTP mRNA is significantly improved upon overexpression of AGO2. 1.5 × 10^4^ cells were simultaneously transduced with (i) a rAdV expressing HAdV-5-targeting (pTP-mi5) or non-targeting (NT) amiRNAs and (ii) a rAdV expressing AGO2 or EGFP at an MOI of 250, before being exposed to wt HAdV-5 at an MOI of 1. Concentrations of wt HAdV-5 genome copy numbers were measured at 72 h post-infection using an E3-probe qPCR. Each experiment’s EGFP control preparation measurement was set as a reference at 100%. The inhibition by AGO2 alone in the absence of any targeting or non-targeting amiRNA is shown for comparison. Data were derived from a total of 3 representative experiments and display mean ± standard deviations. * (*p* < 0.05); ns (not significant). AGO2-mediated differences between 6xNT and 6xpTP-mi5 were significant in all instances. (**B**) Evaluation of the impact of adenoviral vector MOIs in relation to wt HAdV-5 MOIs on viral replication and vector mobilization. 1.5 × 10^4^ HeLa cells were simultaneously transduced with rAdVs at MOIs as per the X-axis and infected with wt HAdV-5 as per the Y-axis. Concentrations of wt HAdV-5 and vector genome copy numbers were measured at 48 h post-infection using E3- and CMV promoter-specific qPCR probes, respectively.

As ectopic expression of AGO2 leads to an impairment of adenoviral replication, an effect likely—to at least some extent—owing to increased targeting of viral transcripts by endogenous miRNAs or their respective mimics, the same should consequently apply to amiRNAs directed against adenoviral transcripts. Such amiRNAs have previously been developed and shown to successfully inhibit HAdV-5 replication both in vitro and in vivo [34,35,36]. We hypothesized that functionally extending our existing therapeutic anti-adenoviral amiRNA vectors [34] by replacing EGFP with AGO2 would further improve their performance. These vectors are based on an E1A/E3-deleted, replication-deficient HAdV-5 backbone and carry six tandemly arranged anti-pTP (pTP-mi5) or control (non-targeting; NT) amiRNAs (Figure 1). Initial co-transduction experiments combining these vectors with either EGFP- or AGO2-encoding rAdV vectors (Figure 1) led to a significant gain of function upon AGO2 overexpression (Figure 6A). This observation resulted in the generation of novel rAdV vectors that combined amiRNA with AGO2 expression (Figure 1). The functionality of the expression cassettes was proven by Western blotting (Figure S3). To assess the reciprocal interplay between rAdV vectors and wt HAdV-5 and identify the appropriate vector dosage, we performed inhibition assays covering a 2D matrix consisting of dilution gradients of vector and wt HAdV-5 MOIs, with the wt genome copy number as the reportable value (Figure 6B). The wt HAdV-5 MOI was investigated across five orders of magnitude (0.01–100), whereas the therapeutic vector MOIs ranged from 50 to 1000. We separately monitored the performance of rAdV vectors derived from previous studies co-expressing EGFP and the novel AGO2-generating vector species. In addition to assessing the HAdV-5 genome copy number, we quantified vector mobilization.

Notably, concerning EGFP-expressing rAdV vectors, we could demonstrate that the degree of wt HadV-5 inhibition sharply revolved around a magnitude of 13.7% (SD ± 1.75%) residual infectious wt virus, irrespective of the wt HAdV-5/vector ratio (Figure 6B). The experimental scheme was designed such that wt HAdV-5 and the vector were added simultaneously and should be able to compete for cellular entry as a function of their relative ratio owing to their equivalent tropism, which is only limited by host cell resources. Referring to vector mobilization, the amplification of the non-targeting control vector was substantially higher than that of its pTP-targeting counterpart (Figure 6B).

This held conceptionally true for the AGO2-expressing novel genotypes. However, the AGO2 vectors displayed a uniformly superior performance with an average fold improvement factor of 7.7 (SD ± 3.3) within the evaluable vector MOI range of 50–100 (Figure 6B). Notably, wt HAdV-5 inhibition revolved around an average of 1.85% (SD ± 0.4%) of the remaining infectious particles. Beyond the above-mentioned vector MOI range of 50–100, no wt HAdV-5 genome replication could be detected in samples treated with the pTP-targeting AGO2 vector, thus preventing appropriate quantification of the de facto inhibition potency. In addition to the systematically improved HAdV-5 inhibition capability, AGO2 vector mobilization was drastically reduced in both non-targeting and pTP-targeting species (Figure 6B). In fact, AGO2/pTP amiRNA vector mobilization was virtually nonexistent across almost three-quarters of the observed conditions.

Eventually, an all-embracing assessment of the anti-adenoviral performance of the AGO2-co-expressing amiRNA vectors over time was conducted using a representative vector/wt HAdV-5 ratio of 100/0.1. In two independent experimental approaches, HeLa cells were transduced and infected using two distinct protocols. A (i) prophylactic scenario includes priming of the cells with a vector before adding wt HAdV-5, whereas the simultaneous addition of virus and vector simulated a (ii) therapeutic approach. Cultures were analyzed over six days to determine the concentrations of wt HAdV-5 infectious particles at time points 0, 2, 4, and 6 as an endpoint.

**Figure 7:**
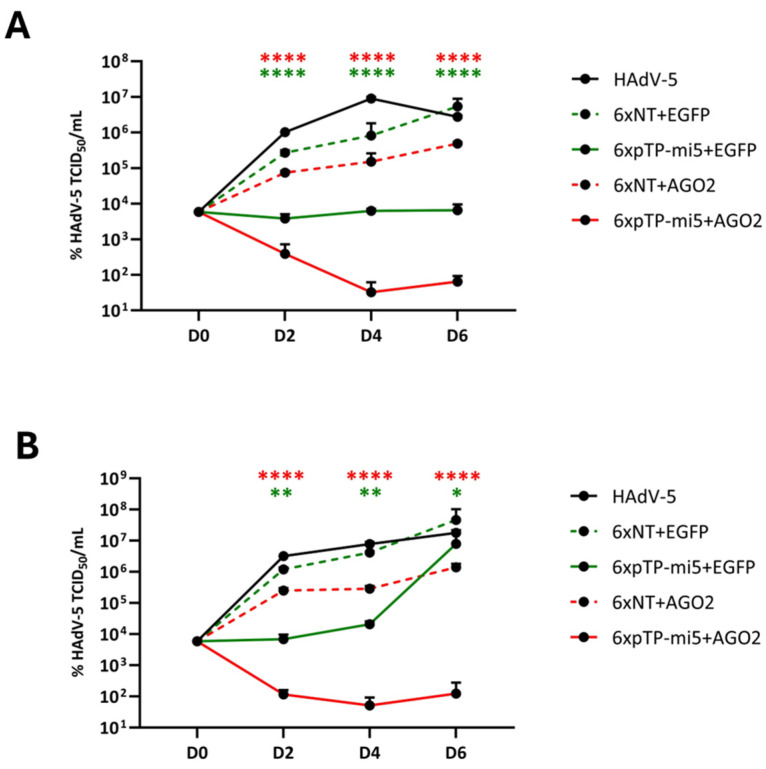
Improvement of rAdV amiRNA vector performance by inclusion of an AGO2 expression cassette. (**A**) In a prophylactic treatment scenario, 1.5 × 10^4^ HeLa cells were transduced with vectors at an MOI of 100 24 h prior to infection with HAdV-5 at an MOI of 0.1. Concentrations of wt HAdV-5 infectious particles (TCID50/mL) were measured at timepoints 0 (D0), 48 h (D2), 96 h (D4), and 144 h (D6). Data were derived from a total of 3 experiments and display mean ± standard deviations. **** (*p* < 0.0001). Significances in green: 6xNT+EGFP vs. 6xpTP-mi5+EGFP; significances in red: 6xNT+AGO2 vs. 6xpTP-mi5+AGO2. (**B**) In a therapeutic treatment scenario 1.5 × 10^4^ HeLa cells underwent concomitant transduction and infection with vectors at an MOI of 100 as well as HAdV-5 at an MOI of 0.1, respectively. Concentrations of wt HAdV-5 infectious particles (TCID50/mL) were measured at timepoints 0 (D0), 48 h (D2), 96 h (D4), and 144 h (D6). Data were derived from a total of 3 experiments and display mean ± standard deviations. **** (*p* < 0.0001); ** (*p* < 0.01); * (*p* < 0.05). Significances in green: 6xNT+EGFP vs. 6xpTP-mi5+EGFP; significances in red: 6xNT+AGO2 vs. 6xpTP-mi5+AGO2.

We showed that AGO2-co-expression leads to markedly improved performance under all tested conditions. Regarding the prophylactic approach, differences across the NT and targeting vector species were highly significant for both EGFP- and AGO2-expressing rAdVs at all time points (Figure 7A). While the targeting EGFP vectors reduced the number of infectious HAdV-5 particles to an extent similar to that observed earlier by us with this type of vector (up to approximately 2.5 orders of magnitude) [34], co-expression of AGO2 significantly boosted the degree of inhibition. When compared to the non-targeting AGO2 control vector, specific inhibition rates improved by 0.46 (D2), 1.55 (D4), and 1.25 (D6) orders of magnitude, respectively, resulting in a reduction of infectious virus particle numbers by up to approximately 4 orders of magnitude. In summary, the improvement in performance by means of the overall inhibition rate of the novel AGO2 vectors was statistically significant (*p* < 0.05) across all time points.

The AGO2 vector-mediated gain-of-function was similarly pronounced in the therapeutic scenario, in which rAdVs and HAdV-5 were simultaneously added to HeLa cell cultures (Figure 7B). Although the EGFP vector pair performance was still statistically significant, p values decreased with the advancing duration of the experiment. Conversely, the inhibition of HAdV-5 by the AGO2 vectors remained significant at the highest level, similar to that observed in experiments using the prophylactic approach (Figure 7A). Likewise, the specific inhibition rates improved by 1.08 (D2), 1.73 (D4), and 1.62 (D6) orders of magnitude, respectively. Additionally, the overall performance improvement of the AGO2 vectors across all time combined was statistically significant (*p* < 0.05). The number of infectious virus particles was decreased by the targeting AGO2 vector by approximately four orders of magnitude compared to the non-targeting AGO2 control vector.

## 4. Discussion

Besides having other functions, eukaryotic RNAi has been recognized as a prominent defense mechanism against non-self RNA [43,44]. This includes antiviral functions, resulting in an ongoing arms race involving viral escape strategies [28,29]. One of these is the production of mivaRNAs by adenoviruses [12].

Adenoviral mivaRNAs have been attributed multiple functions: besides their role in sequence-dependent targeting of specific cellular RNAs [13,16,22] to promote the viral life cycle, they may be pivotal for inhibiting the cellular RNAi machinery in a sequence-independent manner by partially depleting cellular miRNAs from RISCs [14,15,16,21]. At least two types of positive effects for the virus are conceivable: (i) impairment of the ability of endogenous miRNAs to regulate host cell gene expression, creating a beneficial environment for viral replication; and (ii) prevention of targeting viral mRNAs by host miRNAs to promote the production of viral proteins.

In this study, we investigated whether the inherent potency of cellular miRNAs, if not blocked by the virus, would actually be sufficient to exert a significant negative impact on adenovirus replication. Indeed, initial experiments indicated that overexpression of AGO2 led to the inhibition of HAdV-5 replication, as indicated by decreased viral genome copy numbers (Figure 2A,D) and the number of infectious virus particles (Figure S1), likely due to an increasing abundance of functional RISCs. Notably, viral genome copy numbers increased by approximately 2-fold compared to wild-type AGO2 when slicing-deficient AGO2 mutants were overexpressed instead (Figure 2C), suggesting that the detrimental impact on the wt HAdV-5 life cycle, at least mostly, depends on the well-documented ability of AGO2 to physically disintegrate target mRNA and not on alternative functions of AGO2 that have also been described [45]. In this regard, our data are reminiscent of the similar effect of AGO2 overexpression on rotavirus replication, which was reversed upon exposure to the slicing-deficient AGO2 mutants D597A and D669A [46]. In contrast, AGO2 did not affect the HAdV-5 mutant dl-sub720, which lacks the VA RNA I and II loci (Figure 2B), and consequently, mivaRNA production. These data support the hypothesis that mivaRNAs are required to generate a shortage of functional RISCs and that excess or decoy AGO2 derepresses this state, thus making newly available RNAi effector complexes available for other miRNA species. In summary, the mivaRNA function of saturating RISC-residing AGO2 proteins to impede endogenous miRNA-mediated RNAi appears to be beneficial for adenovirus replication.

We hypothesized that de-repressed or reinstated targeting of viral transcripts by host cell miRNAs contributed to the inhibitory effect caused by overexpression of AGO2. In fact, we identified several HAdV-5 mRNAs that contained putative, high-scoring, target sites of cellular miRNAs (Table 1). Dual-luciferase RNAi reporter assays clearly demonstrated that the viral DNA polymerase mRNA was targeted by at least one cellular miRNA, hsa-miR-7. Hsa-miR-7 reduced the readout of the assay by approximately 50% in a statistically significant way (Figure 4B). Efficient targeting of the viral DNA polymerase sequence by hsa-miR-7 was not unexpected, as in silico analyses predicted even two high-scoring putative target sites. Moreover, these sites are located at a distance from each other, which may promote cooperative hsa-miR-7 binding, as described for other miRNAs, resulting in an overall enhanced knockdown of gene expression [47,48,49]. While technically not significant, the performance of other miRNA mimics suggested anti-adenoviral activity. This includes miRNAs hsa-423-5p/3p, -151a-5/3p, -29a-3p, and -let-7a-5p (Figure 4B). Likewise, although we were unable to detect a statistically significant reduction in HAdV-5 preterminal protein (pTP) luciferase signals, hsa-29a-3p, -27b-3p, -21-5p, and -7-5p consistently showed a tendency to affect the readout to some degree (Figure 4C).

We confirmed these results by reversing the effect of miRNA mimics with PIs binding to the targeting (guide) strand of the respective miRNA mimic duplex (Figure 5A). Beyond PI-7-5p, PI-423-5p showed a similar pattern, resulting in an increase in the signal upon PI exposure, whereas PI-423-3p had no substantial effect. This observation, although not statistically significant, showed the same trend as seen during PI-7-5p-mediated approaches and suggested that hsa-miR-423-5p may be the effector miRNA strand, whereas the -423-3p species would not be preferentially incorporated into RISC complexes. Importantly, the PI targeting hsa-miR-7-5p significantly increased HAdV-5 Pol luciferase signals on its own in the absence of the hsa-miR-7 mimic, suggesting that neutralization of the respective endogenous miRNA species was sufficient to de-repress its background activity (Figure 5B). This suggests that endogenous hsa-miR-7 levels were sufficiently high to allow effective targeting of the DNA polymerase sequence. It may be appropriate to conclude that hsa-miR-7, if not indirectly hindered by virus-derived mivaRNAs, would indeed be sufficiently potent to significantly knock down viral DNA polymerase gene expression during infection. Because hsa-miR-7 generated the most pronounced effect, we focused on this particular miRNA. However, the cumulative action exerted by all endogenous miRNAs (including those not tested here) targeting this particular adenoviral transcript may be synergistic and more pronounced than a single action. Moreover, other viral transcripts must be expected to be subject to targeting by individual and collective actions of endogenous miRNAs, thus further impacting overall viral replication.

In summary, although cellular miRNAs, such as hsa-miR-7, may have an additional, indirect, inhibitory effect on viral replication by additionally targeting host cell transcripts, leading to a net benefit for the virus, our luciferase assay data support the hypothesis that one role of mivaRNAs may indeed be to protect adenoviral RNAs from becoming targets of endogenous miRNAs. Eventually, not only the saturation of AGO2 proteins but also other proteins exerting functions upstream of RISC incorporation of miRNAs in the RNAi pathway are likely to contribute to the overall impairment of RNAi in adenovirus-infected cells. This includes the processing of miRNA precursors and their export from the nucleus, as previously described [14,18,50].

The fact that the mivaRNA-mediated bottleneck concerning functional RISCs could be alleviated by ectopic overexpression of AGO2, thus rendering the viral mRNAs more susceptible to host cell miRNAs, should also have consequences for therapeutic approaches aimed at inhibiting adenoviral replication by amiRNAs [34,35,36].

Although ectopic overexpression of AGO2 may have established a generally negative environment for virus replication in our experiments, as observed during overexpression of AGO2 in the absence of any targeting or non-targeting amiRNAs, it notably improved the power of pTP-targeting amiRNAs (Figure 6 and Figure 7): when HAdV-5 was exposed to generation 1 adenoviral vectors co-expressing AGO2 and hexameric, tandemly arranged pTP-targeting amiRNAs, it could barely replicate its genome or generate infectious particles. Notably, the anti-adenoviral effect of AGO2 was surprisingly constant across a multitude of inhibition assay conditions, whereas the factual AGO2-mediated improvement itself ranged from 0.57 to 1.19 orders of magnitude. Hence, this novel anti-adenoviral vector generation displayed superior antiviral performance at decidedly low doses compared to the former generation expressing amiRNAs in the absence of AGO2 [34] (Figure 7). While it is challenging to deconvolute the vector-mediated wt HadV-5 inhibition and attribute it to either the isolated impact of AGO2 or an amiRNA/AGO2-derived gain of function, the numbers suggest that an approximate four-fold reduction by the former and a two-fold decrease by the second parameter add up to an eight-fold reduction in wt HadV-5 genome copy numbers, as suggested by the HeLa matrices.

In this study, we used replication-deficient HAdV-5-based vectors for the delivery of amiRNAs and AGO2. These vectors are capable of infecting the same cell types as the wild-type virus, which may help in possible therapeutic applications. They lack central genes (E1, E3) required for viral replication which renders them replication-deficient in uninfected cells. However, upon encounter with wt adenovirus which can provide the required gene products in trans the vectors start to replicate. In this way, the amiRNA and AGO2 gene copy numbers and consequently amiRNA and AGO2 levels are selectively increased in HAdV-5-infected cells. This specific feature may also be beneficial in a therapeutic application. It may also help to spread the vector at the site of infection. The same principle would apply to adeno-associated virus (AAV) vectors, which also need the presence of adenovirus for replication [51]. Such delivery systems can be expected to be self-balancing to some extent; in cases where inhibition of HAdV-5 is very poor, the vector will start to replicate, thereby leading to the production of higher levels of anti-adenoviral components. While we saw significant replication of vectors carrying only amiRNAs in HAdV-5-infected cells (the previous generation of amiRNA vectors; [34]), the concomitant expression of AGO2 strongly decreased the replication of the vector (Figure 6B), underscoring its improved performance. However, the ability to replicate if the wt adenovirus is not efficiently blocked may become important in other cell types or in vivo.

Future work will clarify whether the combination of significantly improved inhibition capacity at low therapeutic vector doses would favor improved and/or accelerated clearance of the virus from an infected organism in vivo. Owing to their rather straightforward construction procedure, amiRNAs have great potential for application as therapeutic agents, the scope of which may extend beyond inhibiting adenovirus replication.

## 5. Conclusions

Human AGO2, a rate-limiting RISC component, negatively interferes with the life cycle of HAdV-5 by inhibiting genome amplification. In addition, there is evidence that endogenous miRNAs are involved in mechanisms to impair HAdV-5 replication, with hsa-miR-7-5p and -423-5p/3p having the most distinctive effects. When mivaRNA-mediated depletion of endogenous miRNAs from RISCs is reversed by overexpression of AGO2, these miRNAs regain their ability to target viral mRNAs, making the virus more vulnerable to cellular RNAi. Likewise, amiRNAs specific for adenoviral transcripts also become more effective in inhibiting viral replication, suggesting a great benefit of co-expression of amiRNAs and AGO2 in strategies for the therapeutic inhibition of adenovirus replication.

## Figures and Tables

**Table 1:** Mimics of cellular miRNAs and their putative target sites.

No.	hsa-miR-	TargetScan Score	5p [5′→3′]	3p [5′→3′]	Target Site 5′	Target Site 3′	Target Site Characteristic
1	let-7a-5p	−530	UGAGGUAGUAGGUUGUAUAGUU	CUAUACAAUCUACUGUCUUUC	4229	4251	pTP+Pol 3′UTR
	let-7a-5p	−626			9120	9142	pTP ORF
	let-7a-5p	−428			8612	8634	pTP ORF
2	miR-100-5p	−698	AACCCGUAGAUCCGAACUUGUG	CAAGCUUGUAUCUAUAGGUAUG	6258	6280	Pol ORF/pTP 3′UTR
3	miR-125b-5p	−1158	UCCCUGAGACCCUAACUUGUGA	ACGGGUUAGGCUCUUGGGAGCU	4990	5012	pTP+Pol 3′UTR
4	miR-1307-5p	−1755	UCGACCGGACCUCGACCGGCU	ACUCGGCGUGGCGUCGGUCGUG	9680	9702	pTP ORF
5	miR-151a-3p	−526		CUAGACUGAAGCUCCUUGAGG	10,154	10,176	pTP ORF
	miR-151a-5p	−738	UCGAGGAGCUCACAGUCUAGU		6151	6173	Pol ORF/pTP 3′UTR
	miR-151a-5p	−610			6843	6865	Pol ORF/pTP 3′UTR
6	miR-22-3p	−581	AGUUCUUCAGUGGCAAGCUUUA	AAGCUGCCAGUUGAAGAACUGU	4978	5000	pTP+Pol 3′UTR
7	miR-27b-3p	−465	AGAGCUUAGCUGAUUGGUGAAC	UUCACAGUGGCUAAGUUCUGC	9721	9743	pTP ORF
8	miR-29a-3p	−1138	ACUGAUUUCUUUUGGUGUUCAG	UAGCACCAUCUGAAAUCGGUUA	4234	4256	pTP+Pol 3′UTR
	miR-29a-3p	−583			9850	9872	pTP ORF
9	miR-423-3p	−707		AGCUCGGUCUGAGGCCCCUCAGU	5182	5204	Pol ORF/3′UTR+ pTP 3′UTR
	miR-423-3p	−702			7965	7987	Pol ORF/pTP 3′UTR
	miR-423-3p	−698			5733	5755	Pol ORF/pTP 3′UTR
	miR-423-3p	−713			10,265	10,287	pTP ORF
	miR-423-5p	−737	UGAGGGGCAGAGAGCGAGACUUU		6751	6773	Pol ORF/pTP 3′UTR
	miR-423-5p	−657			5528	5550	Pol ORF/pTP 3′UTR
	miR-423-5p	−656			10,374	10,396	pTP ORF
10	miR-7-5p	−1113	UGGAAGACUAGUGAUUUUGUUGUU	CAACAAAUCACAGUCUGCCAUA	6942	6964	Pol ORF/pTP 3′UTR
	miR-7-5p	−559			6888	6910	Pol ORF/pTP 3′UTR
	miR-7-5p	−1011			9545	9567	pTP ORF
11	miR-99b-5p	−694	CACCCGUAGAACCGACCUUGCG	CAAGCUCGUGUCUGUGGGUCCG	6258	6280	Pol ORF/pTP 3′UTR

## Data Availability

The most relevant datasets generated and analyzed as part of this study are included in the article/Supplementary Material, and further inquiries can be directed to the corresponding author.

## Supplementary Materials

The following supporting information can be downloaded at: https://www.mdpi.com/article/10.3390/cells13131117/s1, Figure S1: Human AGO2 impairs the production of infectious wt HAdV-5 particles; Figure S2: rAdV-delivered AGO2 negatively affects HAdV-5 replication across a multitude of vector MOIs, transduction/infection scenarios, and independent vector preparations. Figure S3: Evaluation of the functionality of the AGO2 expression cassettes present in plasmid and adenoviral vectors in terms of AGO2 overexpression.
