# Supplementary material for: Anti-Adenoviral Effect of Human Argonaute 2 Alone and in Combination with Artificial microRNAs
Source: Cells. 2024 Jun 28;13(13):1117. doi: 10.3390/cells13131117 (PMC11240694; doi:10.3390/cells13131117)
Supplement: Supplementary file 1 [file cells-13-01117-s001.zip › cells-3053554-supplementary.pdf]

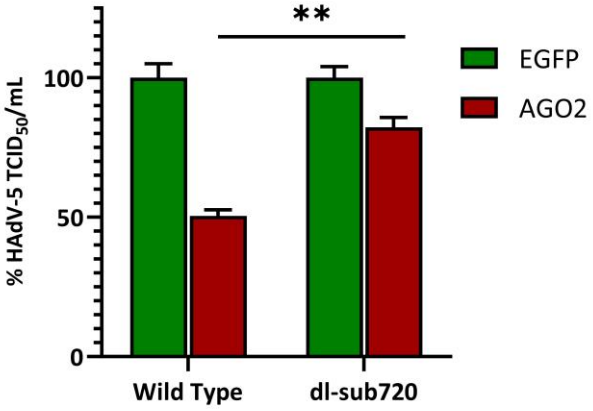

Figure S1. Human AGO2 impairs the production of infectious wt HAdV-5 particles. 1.5E+04 HeLa cells were simultaneously transfected with 250 ng EGFP- or AGO2-expressing plasmid and infected with HAdV-5 or HAdV-5 mutant dl-sub720 at an MOI of 0.1, respectively. Concentrations of HAdV-5/dl-sub720 infectious particles (TCID50/mL) measured at 96h post-infection (means ± standard deviations) are shown. ** (p<0,01).

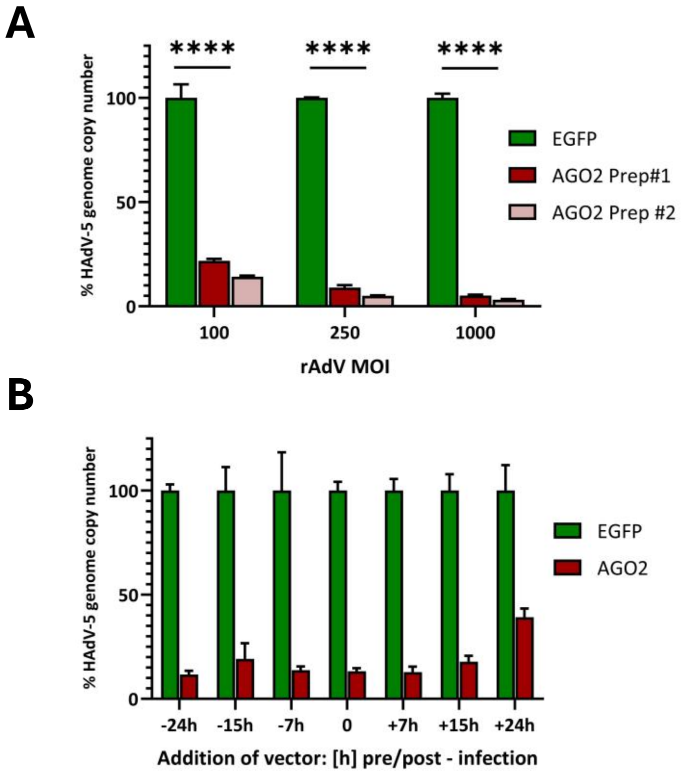

Figure S2. rAdV-delivered AGO2 negatively affects HAdV-5 replication across a multitude of vector MOIs, transduction/infection scenarios, and independent vector preparations. (**A**) 1.5E+04 A549 cells were transduced with rAdV vectors as per indicated MOIs for 24h. After an 8h washing step, wt HAdV-5 was added at an MOI of 1. Two independently prepared and characterized AGO2-expressing rAdV vectors were run side by side. Concentrations of HAdV-5 genome copy numbers were measured at 48h post infection using a E3-probe qPCR. Data represent means ± standard deviations of a representative experiment. **** ($p<0.0001$). (**B**) The efficiency in downregulation of HAdV-5 replication by AGO2-expressing rAdV when applied before or after infection with wt HAdV-5 was assessed by transduction of cells with the rAdVs at an MOI of 250 at the indicated time points before or after infection with HAdV-5 at an MOI of 0.1. Data represent means ± standard deviations of a representative experiment. Each timepoints' AGO2 performance was statistically significant ($p<0.0001$).

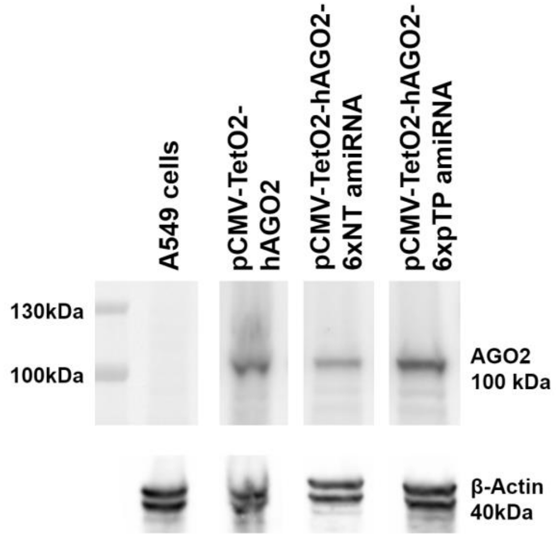

Figure S3. Evaluation of the functionality of the AGO2 expression cassettes present in plasmid and adenoviral vectors in terms of AGO2 overexpression. Briefly, 1.25E+05 A549 cells were seeded into 24-wells before being transduced with adenoviral vectors carrying the expression cassettes for AGO2 expression alone or for concomitant expression of non-targeting (NT) or pTP-targeting amiRNAs at an MOI of 100. Mock-transduced A549 cells served as a control. Cells were harvested 48 h post-transduction and were analysed by sodium dodecyl sulphate-polyacrylamide gel electrophoresis followed by Western blotting using an anti-AGO2 antibody. β-actin staining was performed for comparison.
